# Investigation of Surface Integrity of Selective Laser Melting Additively Manufactured AlSi10Mg Alloy under Ultrasonic Elliptical Vibration-Assisted Ultra-Precision Cutting

**DOI:** 10.3390/ma15248910

**Published:** 2022-12-13

**Authors:** Rongkai Tan, Xuesen Zhao, Qi Liu, Xianmin Guo, Fengtao Lin, Liquan Yang, Tao Sun

**Affiliations:** 1 Key Laboratory of Conveyance and Equipment of Ministry of Education, East China Jiaotong University, Nanchang 330013, China; 2 Henan Province Engineering Research Center of Ultrasonic Technology Application, Pingdingshan University, Pingdingshan 467000, China; 3 Jiangxi Province Engineering Research Center of Drug and Medical Device Qualit, NMPA Key Laboratory of Quality Evaluation of Traditional Chinese Patent Medicine, Jiangxi Institute for Drug Control, Nanchang 330029, China; 4 Center for Precision Engineering, Harbin Institute of Technology, Harbin 150001, China; 5 Department of Design, Manufacturing and Engineering Management, University of Strathclyde, Glasgow G1 1XQ, UK

**Keywords:** ultrasonic elliptical vibration-assisted cutting, ultra-precision cutting, additively manufactured AlSi10Mg alloy, surface integrity, tool wear

## Abstract

Additive manufacturing technology has been widely used in aviation, aerospace, automobiles and other fields due to the fact that near-net-shaped components with unprecedented geometric freedom can be fabricated. Additively manufactured aluminum alloy has received a lot of attention, due to its excellent material properties. However, the finished surface of additively manufactured aluminum alloy with nanoscale surface roughness is quite challenging and rarely addressed. In this paper, a novel machining technology known as ultrasonic elliptical vibration-assisted cutting (UEVC) was adopted to suppress the generation of cracks, improve the surface integrity and reduce tool wear during the ultra-precision machining of selective laser melting (SLM) additively manufactured AlSi10Mg alloy. The experimental results revealed that, in the conventional cutting (CC) process, surface defects, such as particles, pores and grooves, appeared on the machined surface, and the machined surface rapidly deteriorated with the increase in cumulative cutting area. In contrast, an almost flawless machined surface was obtained in the UEVC process, and its roughness value was less than 10 nm. Moreover, the tool wear of the CC tool was remarkably greater than that of the UEVC tool, and the standard flank wear width of the CC tool was more than twice that of the UEVC tool. Therefore, the UEVC technology is considered to be a feasible method for the ultra-precision machining of SLM additively manufactured AlSi10Mg alloy.

## 1. Introduction

Additive manufacturing technology is able to manufacture near-net-shaped parts with unprecedented geometric freedom, which is considered promising [1,2]. Selective laser melting (SLM) is one of the main additive manufacturing techniques, in which the metallic parts can be directly created by melting metal powder [3]. Additively manufactured parts have been widely used in aviation, aerospace, automobiles and other fields due to their excellent economic benefits and huge application potential. However, the additive manufacturing methods also have some disadvantages, such as incomplete powder melting, increased porosity, high surface roughness and unsatisfactory dimensional and shape accuracy, which lead to the fact that the additively manufactured parts are usually not directly usable, especially in precision and ultra-precision applications [4,5,6]. Therefore, an additional machine-finishing step is usually required for achieving close tolerances and improving the surface quality. However, notably, the properties of the additively manufactured material significantly differ from those of the traditional forged material due to the complex heat transfer of the material during the laser melting and cooling processes [1,7,8]. The additively manufactured material is usually accompanied by large and highly inhomogeneous residual stresses, enhanced strength and toughness and unmelted or only partially melted powder particles; therefore, the machinability of additively manufactured parts is significantly different from that of wrought or cast metals [4,9].

Additively manufactured aluminum alloy has received a lot of attention, and it is frequently used in the aerospace, aviation, shipbuilding and optical engineering fields because of its excellent mechanical properties, such as, light weight, high strength, corrosion resistance and small thermal expansivity. In recent years, intensive research has been carried out to understand the physical principles and to study the machinability of additively manufactured aluminum alloys [10,11,12]. Struzikiewicz et al. [13] researched the machinability of SLM additively manufactured AlSi10Mg alloy; their findings indicated that breaches appeared on the finished surface, which adversely affected the value of surface roughness. In addition, breaches, pores and failure-like cracks were also found on the finished surface in the milling of SLM additively manufactured AlSi10Mg alloy, and the recommended machining method was down-milling [14]. The thrust forces during the drilling machining of additively manufactured and conventionally wrought AlSi10Mg were examined by Ullah et al. [6]. Their findings indicated that the thrust forces could be tested by drilling machining of the wrought material and were obviously smaller than those in the additively manufactured material. Zimmermann et al. [4] studied chips, cutting forces, surface morphology, micro-hardness and burr formation during the milling of conventionally cast and additively manufactured AlSi10Mg aluminum alloy. Guo et al. [15] investigated the ultra-precision machining performance of V-groove structures on additively manufactured RSA-905 alloy. The surface roughness (Ra) of the machined surface was 15nm under the best machining condition, which indicated that the ultra-precision machinability of additively manufactured aluminum alloy was poor compared with that of wrought or cast aluminum alloy. Moreover, experimental research on the magnetic field-assisted machining of additively manufactured RSA-905 alloy was carried out by Guo et al. [16]. The above studies indicated that the machinability of additively manufactured aluminum alloy was worse in comparison to the conventional wrought or cast metals, and the finished surface of additively manufactured aluminum alloy with nanoscale surface roughness was a serious challenge and was rarely resolved. Hence, a better machining method is necessary to improve the ultra-precision machinability of additively manufactured aluminum alloy.

Ultrasonic elliptical vibration-assisted cutting (UEVC) is a promising machining technology that is particularly advantageous compared to conventional cutting (CC), such as a smaller cutting force, extended tool life, better cutting stability and improved surface integrity [17,18,19,20]. Furthermore, the ultra-precision machined surfaces of difficult-to-machine materials were obtained by using UEVC technology with a diamond tool [21,22,23,24,25,26,27]. It should be noted that, during the UEVC machining process, the extrusion effect exerted by the cutting tool is periodically applied to the machined surface, which causes the inhibition of the generation of pores and particles [26]. In addition, the smaller cutting and friction forces are beneficial to suppress the generation of cracks, reduce the residual stress and improve the machined surface’s quality [27,28]. However, a comprehensive investigation of the ultra-precision machining of SLM additively manufactured AlSi10Mg alloy has not been performed using UEVC technology.

Thus, a comprehensive investigation of the feasibility of UEVC technology as a machining method for the ultra-precision cutting of SLM additively manufactured aluminum alloy is needed. In this paper, the influence mechanism of UEVC technology regarding the suppression of the generation of cracks, the improvement of surface integrity and the suppression of tool wear is considered during the ultra-precision machining of SLM additively manufactured aluminum alloy. The structure of this article is as follows. First of all, the UEVC principles and the experimental setup are presented. Then, the comprehensive investigation of surface integrity and tool wear during UEVC and CC processes is performed. Finally, the conclusions are drawn. In this paper, a feasible machining method is presented for the ultra-precision cutting of SLM additively manufactured aluminum alloy.

## 2. Materials and Methods

### 2.1. The UEVC Principle

**Figure 1:**
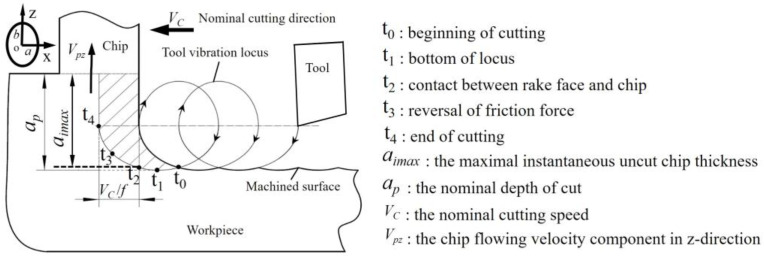
Schematic diagram of UEVC.

As shown in Figure 1, in the UEVC cutting process, in addition to the normal cutting motion, the tool experiences periodic vibration in both the nominal cutting depth direction (i.e., *z*-axis) and nominal cutting direction (i.e., *x*-axis). The tool vibration amplitudes in both directions are in the micron scale. The trajectory of the tool tip can be described as follows:

(1) $$\{\begin{array}{c} x(t)=a\cos\left(2\pi ft\right)-V_{C}t\\ z(t)=b\cos\left(2\pi ft+\varphi \right) \end{array}$$

where *a* and *b* represent the vibration amplitudes in the cutting and cutting depth directions. *f* represents the vibration frequency. *φ* represents the phase shift between two vibration signals. Based on Equation (1), the instantaneous velocity of the tool tip can be obtained as follows:

(2) $$\{\begin{array}{c} x^{\prime }(t)=-2\pi fa\sin(2\pi ft)-V_{C}\\ z^{\prime }(t)=-2\pi fb\sin(2\pi ft+\varphi ) \end{array}$$

Remarkably, when *Vc* < 2πfa, the cutting process is intermittent. Moreover, 2πfa is usually set to more than 12 times that of *Vc* during ultra-precision cutting [29].

As displayed in Figure 1, during the UEVC process, the cutting tool passes through five important time points, *t*_0_, *t*_1_, *t*_2_, *t*_3_ and *t*_4_, successively. The cutting tool and the workpiece begin to come into contact during time point *t*_0_. Subsequently, the tool reaches the lowest point of the cutting trajectory at time point *t*_1_. It is worth noting that, during the time period (*t*_1_ − *t*_0_), the machined surface is squeezed by the cutting tool, which results in the inhibition of the generation of pores and particles, leading to a significant improvement in the machined surface’s quality [26]. The instantaneous uncut chip thickness reaches its maximum at time point *t*_2_. Significantly, the maximum value of the instantaneous uncut chip thickness is smaller than the nominal one ($a_{i\max}<a_{p}$). The velocity component of the tool in the z-direction becomes equal to the velocity component of the chip flowing velocity in the z-direction ($z^{\prime }(t_{3})=V_{pz}$) at time point *t*_3_. Moreover, during the time period (*t*_4_ − *t*_3_), the velocity component of the tool in the z-direction is increasing; thus, the friction force between the tool and chip is reversed. Following this, the cutting tool separates from the workpiece at time point *t*_4_. In summary, the intermittent machining and the diminution of the instantaneous uncut chip thickness lead to the enlargement of the cooling effect and the diminution of the cutting and friction forces, leading to a reduced cutting temperature and extended tool life. In addition, the effect of reversed friction results in an increase in the nominal shear angle, which contributes to the significant diminution of the cutting and friction forces. Most notably, the extrusion effect of the cutting tool on the workpiece is beneficial to the inhibition of the generation of pores and particles. Thus, the ultra-precision machinability of SLM additively manufactured AlSi10Mg alloy can be enhanced through UEVC technology.

### 2.2. Experimental Setup

**Figure 2:**
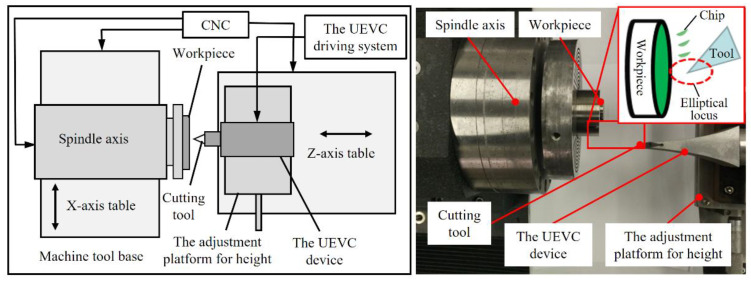
Experimental setup.

**Figure 3:**
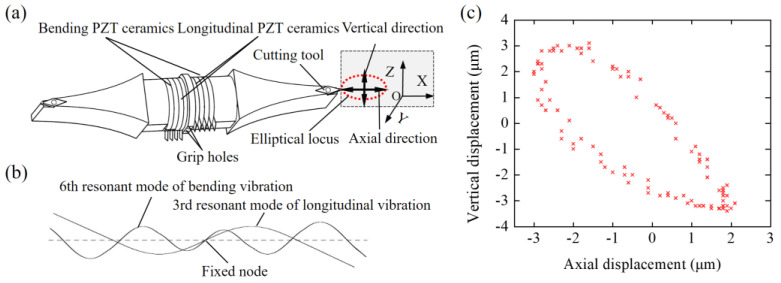
(**a**) A structural drawing of the UEVC device; (**b**) Vibration modes of UEVC device; (**c**) Vibration trajectory of tool tip.

As shown in Figure 2, a home-made ultra-precision machine tool with two horizontal hydrostatic sideways and an aerostatic spindle was used. The workpiece was glued to the adapter, the adapter was held by the vacuum chuck attached on the aerostatic spindle axis, and the aerostatic spindle was assembled on the *x*-axis slideway. The UEVC device was positioned on the high-precision adjustment platform, and it was used to achieve the height adjustment of the UEVC device. Figure 3 shows the elementary diagram of the UEVC device employed in this study [30]. When the UEVC device was not powered, it could be considered as a traditional tool holder. Thus, the UEVC and CC processes could be performed through the UEVC device. A commercial PCD tool (VCGW110310-1N, Beijing Worldia Diamond Tools Co., Ltd., Beijing, China) was employed. In the CC process, the cutting parameters were selected based on the previous literature [15]. In the UEVC process, the spindle speed was selected as 20 r/min to achieve ultra-precise machining, and the values of cutting depth and feed speed were the same as those used in the CC process [26,29]. The experimental parameters are listed in Table 1.

The SLM additively manufactured AlSi10Mg alloy is the most common additively manufactured aluminum alloy, and it was chosen as the workpiece in this study. Table 2 and Table 3 present the physical properties and chemical composition of the selected SLM additively manufactured AlSi10Mg alloy, respectively.

**Figure 4:**
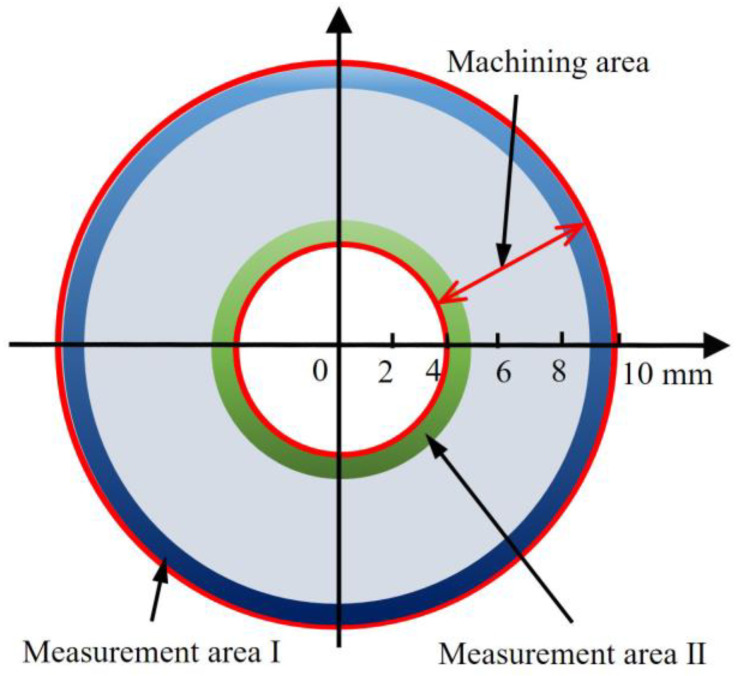
Schematic of machining area and measurement areas.

In this experiment, the workpiece was a round pipe, the height of the workpiece was 10 mm, and the diameter of the workpiece was 20 mm. In this study, the two workpieces were, respectively, used in the CC and UEVC process experiments. The machining surface area was an annular region, as displayed in Figure 4. The inner diameter of the annular region was 8 mm and the outer diameter was 20 mm; thus, the area of the machining surface was around 264 mm^2^. When the cutting experiments were completed, two measurement areas were selected and analyzed, as shown in Figure 4. Atomic force microscopy (AFM, Nanite B, supplied by Nanosurf Ltd., Liestal, Switzerland) was employed for the measurement of the machined surface’s roughness value. The roughness test for each measurement area was repeated five times, and the average of the test results was calculated and recorded. Furthermore, when the cutting experiments were completed, the detection and assessment of tool wear during the CC and UEVC processes were performed by using a scanning electron microscope (SEM, SU8010, supplied by Hitachi High Technologies Corporation, Tokyo, Japan).

## 3. Results and Discussion

### 3.1. Surface Integrity

**Figure 5:**
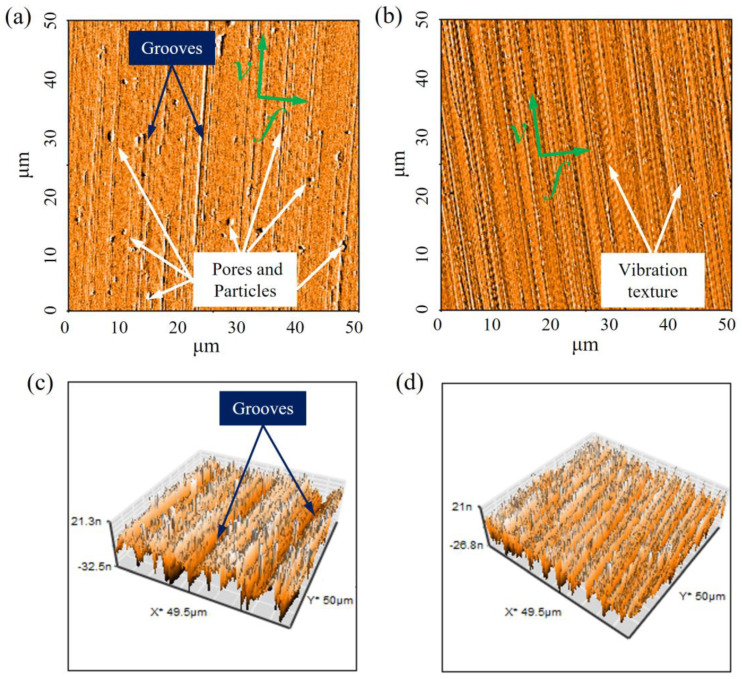
The AFM images of measurement area I: (**a**) two-dimensional machined surface under CC process; (**b**) two-dimensional machined surface under UEVC process; (**c**) three-dimensional machined surface under CC process; (**d**) three-dimensional machined surface under UEVC process.

The two-dimensional and three-dimensional surface topography of the finished surface in measurement area I are shown in Figure 5a,c, respectively, and they were produced by the CC process. Apparently, large amounts of pores and particles were averagely and randomly distributed on the machined surface. This occurred for two possible reasons. First of all, in the selective laser melting process, some particles fail to melt completely, the internal structure of the additively manufactured material is not uniform, and there are small particles and pores. The machining scale of ultra-precision cutting is micron, so these particles and pores easily stand out on the ultra-precision machined surface. The second reason is that the formation of pores and particles is related to the random vibration of the cutting tool in the CC process. Moreover, the inhomogeneity of the additively manufactured material structure is regarded as the main cause of the random vibration of the cutting tool. It is worth noting that grooves also appeared on the machined surface, as shown in Figure 5a,c. This can be explained by the fact that, in the CC process, some particles fail to form chips and slip on the machined surface with the cutting motion of the tool. Furthermore, the small cracks of the cutting tool edge are also a cause of the formation of grooves. Obviously, the surface defects, such as particles, pores and grooves, seriously deteriorated the surface integrity and highly increased the surface roughness value of the finished surface.

In contrast, during the UEVC process, there were no particles, pores and grooves observed on the machined surface, and the vibrational texture was clearly visible in the cutting direction, as illustrated in Figure 5b,d. This is due to the fact that, in the UEVC process, the extrusion effect of the cutting tool on the machined surface is beneficial for the inhibition of the generation of pores and particles, as discussed in Section 2.1. Moreover, the regenerative chatter of the cutting tool is effectively suppressed due to the smaller cutting and friction forces. In addition, during the UEVC process, the regular and clear vibrational texture indicates that the cutting-edge profile is nearly perfect and the machining process is performed smoothly.

**Figure 6:**
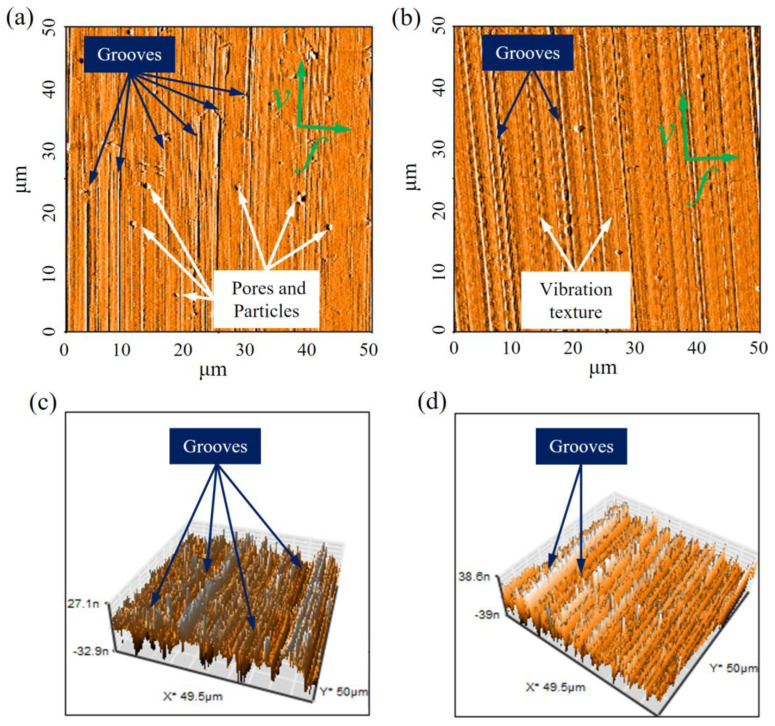
The AFM images of measurement area II: (**a**) two-dimensional machined surface under CC process; (**b**) two-dimensional machined surface under UEVC process; (**c**) three-dimensional machined surface under CC process; (**d**) three-dimensional machined surface under UEVC process.

In the CC process, the number of grooves is visibly enhanced when the cumulative cutting area reaches 264 mm^2^, as shown in Figure 6a,c. Moreover, the pores and particles are still observed on the finished surface. These surface defects lead to a significant deterioration in the machined surface’s quality. It can be speculated that the cutting edge profile of the tool deteriorates further and more notches appear. Moreover, the worn tool further intensifies the random vibration of the cutting tool due to the larger cutting and friction forces. However, as shown in Figure 6b,d, the vibrational texture becomes less noticeable when the cumulative cutting area reaches 264 mm^2^. Additionally, small amounts of grooves with shallow traces appeared on the finished surface. This observation is attributed to the fact that, during the continuous UEVC process, the proper passivation and tiny cracks of the cutting edge of tool occur. However, it is remarkable that the ultra-smooth finished surface was obtained with the passivated tool. The above experimental results indicate that the amount of tool wear is acceptable, and the cutting process is performed smoothly.

**Figure 7:**
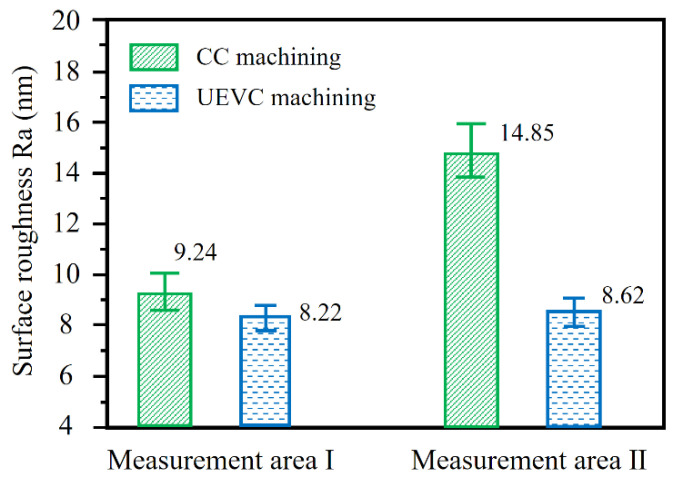
Surface roughness (Ra) values obtained by different machining methods in different measurement areas.

Figure 7 reports the surface roughness (Ra) values obtained by different machining methods in different measurement areas. As expected, the surface roughness value obtained with the UVEC process is lower than that in the CC process. It is worth noting that, in measurement area I, the difference between the roughness values of the finished surface obtained by the CC and UEVC processes is not obvious. This can be interpreted as follows: during the UEVC machining process, the vibrational texture has a detrimental effect on the roughness values of the machined surface. In contrast, during the CC machining process, the surface defects, such as pores, grooves and particles, are the main factors affecting the surface roughness value, as shown in Figure 5a. Furthermore, when the calculation range is set to the AFM test range, namely 50 μm × 50 μm, the influence of surface defects on the surface roughness (Ra) values is similar to that of the vibrational texture on the surface roughness (Ra) value. Moreover, it is worth noting that the repeatability of the surface roughness (Ra) values in measurement area I is better than that in measurement area II. Moreover, the repeatability of the surface roughness (Ra) values with the machined surface produced by the UEVC process is better than that in the CC process.

During the CC process, the surface roughness (Ra) value rapidly grows with the growth in the cumulative machining area. This result is consistent with the previous test results; that is, there are more grooves in measurement area II, which results in a significant increase in the surface roughness values. However, during the UEVC process, the increase in the surface roughness value with the growth in the cumulative machining area is observably lower compared with that in the CC process. It is worth noting that the roughness values of measurement areas I and II are approximately equal and are less than 10 nm. This can be interpreted as follows: during the UEVC machining process, the wear rate of the cutting tool is small. In measurement area II, the negative effect of the grooves on the surface roughness value is roughly equal to the positive effect of the shallower vibrational texture on the surface roughness value. These results indicate that the ultra-precision machined surface of the SLM additively manufactured AlSi10Mg alloy can be obtained through the UEVC technology.

In general, the ultra-precision machined surface of the SLM additively manufactured AlSi10Mg alloy was obtained through the UEVC technology. Another noteworthy fact is that, in the measurement areas, the finished surface exhibited negligible damage, and the machined surface roughness value was less than 10 nm. On the contrary, under the CC machining process, surface defects, such as pores, grooves and particles, emerged on the finished surface, which had an adverse effect on the service performance and service life of the ultra-precision manufactured part. Furthermore, the finished surface was rapidly aggravated with the increase in the cumulative cutting area. Therefore, the experimental results confirm that the UEVC technology plays a great role in improving the surface integrity during the ultra-precision cutting of SLM additively manufactured AlSi10Mg alloy.

### 3.2. Tool Wear

**Figure 8:**
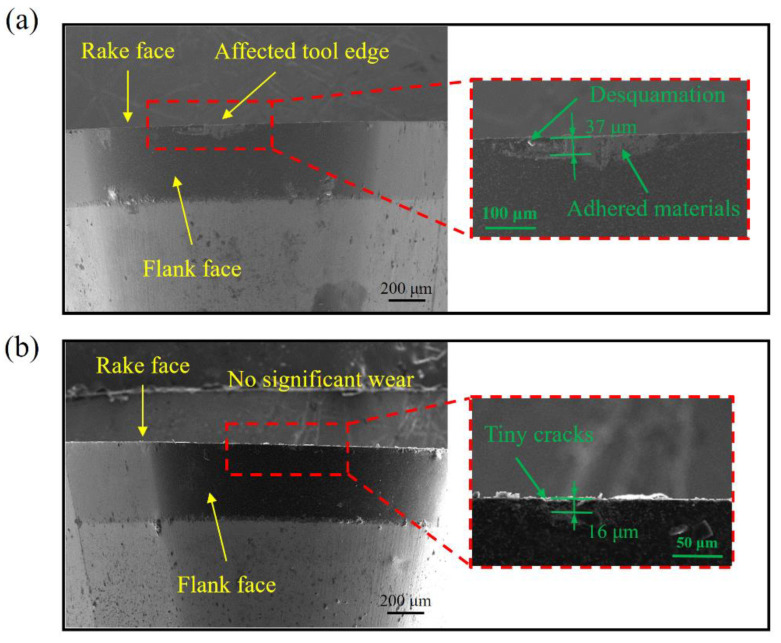
The SEM images of cutting tools under different machining methods: (**a**) the CC tool, (**b**) the UEVC tool.

Figure 8 shows the SEM photographs of the CC tool and UEVC tool, when the cutting experiment was completed. As displayed in Figure 8a, the significant wear of the cutting tool edge was observed. There was significant desquamation on the cutting tool edge and some material bonded to the cutting tool edge. Moreover, according to the standard ISO 3685, the standard flank wear width of the cutting tool was 37 μm. These results indicate that non-negligible wear appeared, which corresponds to the analyzed results of the machined surface. This can be interpreted as follows: the SLM additively manufactured AlSi10Mg alloy has a larger hardness value in comparison to the conventional wrought or cast metals [7,8]. Moreover, during the selective laser melting process, a large number of defects, such as hot cracking and porosity, was generated. Thus, the final properties of the SLM additively manufactured AlSi10Mg alloy were difficult to control. Therefore, during the CC process, the random vibration of the tool and the cutting and friction forces were enhanced, leading to the significantly faster tool wear.

With the same cumulative machining area, the wear of the UEVC tool was obviously smaller than that of the CC tool. As shown in Figure 8b, there was no adhered material, and no obvious wear on the cutting tool edge; only micro-cracks were observed in the further enlargement, which corresponds to the analyzed results of the machined surface. According to the standard ISO 3685, the standard flank wear width of the cutting tool was 16 μm. This can be interpreted as follows: in the UEVC process, the cutting tool is separated periodically from the workpiece in each cutting cycle; the nominal shear angle is increased, and the instantaneous uncut chip thickness is smaller, which result in the remarkable diminution of the friction and cutting forces. Moreover, more remarkably, the extrusion influence of the cutting tool on the workpiece is beneficial to the inhibition of the generation of pores and particles, and the random vibration of the cutting tool is suppressed, which is beneficial in reducing tool wear. These results indicate that, during the ultra-precision cutting of SLM additively manufactured AlSi10Mg alloy, the extension of the tool life was achieved through the UEVC technology.

## 4. Conclusions

The UEVC technology is introduced in the ultra-precision machining of SLM additively manufactured AlSi10Mg alloy to improve the machinability. The surface integrity and tool wear under the CC and UEVC processes were compared and analyzed. Based on the experimental results, some conclusions are proposed.

(1) The experimental results of surface integrity reveal that the UEVC technology plays a great role in improving the surface integrity during the ultra-precision cutting of SLM additively manufactured AlSi10Mg alloy. The surface defects, such as grooves, pores and particles, are averagely and randomly distributed on the machined surface under the CC process. Moreover, the finished surface was rapidly aggravated with the increase in the cumulative cutting area. However, an ultra-precision finished surface of the SLM additively manufactured AlSi10Mg alloy was obtained during the UEVC process. The finished surface exhibited negligible damage, and the machined surface’s roughness value was less than 10 nm. The extrusion effect of the cutting tool on the workpiece, the suppression of the regenerative chatter of the cutting tool, and no obvious wear of the cutting tool edge were the most important factors in improving the surface integrity.
(2) The experimental results of tool wear reveal that, during the ultra-precision cutting of SLM additively manufactured AlSi10Mg alloy, the extension of the tool life was achieved through the UEVC technology. In the CC process, the significant wear of the cutting tool edge was observed, there was significant desquamation on the cutting tool edge, and some material bonded to the cutting tool edge. In contrast, during the UEVC process, there was no obvious wear on the cutting tool edge, no machined material adhered to the flank face, and only tiny cracks were observed in the further enlargement. The lower friction and cutting forces and the smooth cutting process were the most important factors for the suppression of tool wear and the extension of the tool life.
(3) The ultra-precision finished surface of the SLM additively manufactured AlSi10Mg alloy was obtained, and the significant amelioration of surface integrity and suppression of tool wear were achieved simultaneously, indicating that the ultra-precision machinability of SLM additively manufactured AlSi10Mg alloy can be enhanced through UEVC technology. Further research should be conducted to achieve the greater dimensional accuracy of parts by optimizing the machining path of the cutting tool.

## Figures and Tables

**Table 1:** Experimental conditions.

Cutting Method		CC Process	UEVC Process
Vibration parameters	Amplitude in cutting direction (μm)	-	6.5
	Amplitude in cutting depth direction (μm)	-	5
	Frequency (kHz)	-	29.75
	Phase shift difference (°)	-	120
Cutting parameters	Speed (r/min)	1600	20
	Depth of cut (μm)	5	5
	Feed rate (μm/r)	5	5
Cutting tool	Material	Polycrystalline diamond	
	Radius (mm)	1.0	
	Clearance angle (°)	11	
	Rake angle (°)	0	
Workpiece	Workpiece material	Additively manufactured AlSi10Mg alloy	
	Dimension (mm)	*Φ*20 × *L*10	
Coolant		Air cooling	

**Table 2:** Material properties of the selected SLM additively manufactured AlSi10Mg alloy.

Tensile Strength (MPa)	Elastic Modulus (GPa)	Brinell Hardness (HB)	Elongation A5 (%)	Density (g/mm^3^)
270	75	124	3	2.65

**Table 3:** Chemical composition of the selected SLM additively manufactured AlSi10Mg alloy (%).

Si	Mg	Fe	Mn	Ti	Zn	Cu	Ni	Pb	Sn	Al
9.57	0.45	≤0.55	≤0.45	≤0.15	≤0.1	≤0.05	≤0.05	≤0.05	≤0.05	Balance
